# Hypermethylation of GNA14 and its tumor-suppressive role in hepatitis B virus-related hepatocellular carcinoma

**DOI:** 10.7150/thno.48739

**Published:** 2021-01-01

**Authors:** Guangyuan Song, Xingxin Zhu, Zefeng Xuan, Long Zhao, Haijiang Dong, Jian Chen, Zequn Li, Wenfeng Song, Cheng Jin, Mengqiao Zhou, Haiyang Xie, Shusen Zheng, Penghong Song

**Affiliations:** 1 Division of Hepatobiliary and Pancreatic Surgery, Department of Surgery, The First Affiliated Hospital, Zhejiang University School of Medicine; 2 NHC Key Laboratory of Combined Multi-organ Transplantation; 3 Key Laboratory of the diagnosis and treatment of organ Transplantation, Research Unit of Collaborative Diagnosis and Treatment For Hepatobiliary and Pancreatic Cancer, Chinese Academy of Medical Sciences (2019RU019); 4 Key Laboratory of Organ Transplantation, Research Center for Diagnosis and Treatment of Hepatobiliary Diseases, Zhejiang Province, Hangzhou 310003,China

**Keywords:** HCC, DNA methylation, GNA14, HBV, HBx

## Abstract

Hepatocellular carcinoma (HCC) is one of the most lethal cancers worldwide, and its specific mechanism has not been fully elucidated. Inactivation of tumor suppressors may contribute to the occurrence, progression, and recurrence of HCC. DNA methylation is a crucial mechanism involved in regulating the occurrence of HCC. Herein, we aimed to identify the key methylation-related tumor suppressors as well as potential biomarkers and therapeutic targets in HCC.

**Methods**: Combined analysis of TCGA and GEO databases was performed to obtain potential methylation-related tumor suppressors in HCC. Methyl-target sequencing was performed to analyze the methylation level of the GNA14 promoter. The diagnostic value of GNA14 as a predictor of HCC was evaluated in HCC tumor samples and compared with normal tissues. The functional role of GNA14 and its upstream and downstream regulatory factors were investigated by gain-of-function and loss-of-function assays *in vitro*. Subcutaneous tumorigenesis, lung colonization, and orthotopic liver tumor model were performed to analyze the role of GNA14 *in vivo.*

**Results**: The expression of GNA14 was found to be downregulated in HCC and it was negatively correlated with hepatitis B virus (HBV) infection, vascular invasion, and prognosis of HCC. DNA methylation was demonstrated to be responsible for the altered expression of GNA14 and was regulated by HBV-encoded X protein (HBx). GNA14 regulated the RB pathway by promoting Notch1 cleavage to inhibit tumor proliferation, and might inhibit tumor metastasis by inhibiting the expression of JMJD6.

**Conclusion**: GNA14 could be regulated by HBx by modulating the methylation status of its promoter. We identified GNA14 as a potential biomarker and therapeutic target for HCC.

## Introduction

Hepatocellular carcinoma (HCC) remains one of the most aggressive tumors worldwide^1^. Hepatitis B virus (HBV) infection and alcoholic liver disease are major risk factors for the development of HCC^2^. Despite considerable progress in hepatectomy, immunotherapy, and liver transplantation, the prognosis of HCC patients remains poor, which is mainly related to the metastasis and recurrence of HCC 3. Therefore, further elucidation of the key molecular mechanisms behind HCC progression is conducive to the development of new diagnostic and therapeutic strategies.

Guanine nucleotide-binding protein subunit α (GNA) is a member of the G-protein that conducts extracellular signal transduction 4. The GNA protein family has been reported to be involved in the development of HCC. High expression of GNA13 is associated with poor prognosis in HCC 5, while GNAi3 inhibits HCC cell migration and invasion 6. GNA14 has been found to be associated with the development of gastric cancer and vascular tumors 7, 8. However, whether it can affect HCC remains unknown.

HBV infection is one of the dominant predisposing factors for HCC 9. HBV infection can promote the proliferation, angiogenesis, and invasion of HCC 10-13. HBV-encoded X protein (HBx), encoded by covalently closed circular DNA (cccDNA) from HBV, is an essential gene for the emergence of HBV-associated HCC 14. As a multifunctional protein, HBx has been reported to regulate the transcription of target genes by affecting intracellular signal transduction and transcription factors 15, by interfering with the natural function and characteristics of host cells 16.

DNA methylation, an important epigenetic modification, affects the development of tumors in a variety of ways 17-19. Upregulated DNA methylation of tumor suppressive genes leads to their decreased expression, which is a crucial inducement factor for HCC 20. HBV infection has been reported to induce gene methylation in HCC 21. Young et al. and Han et al. demonstrated that HBx enhanced total DNA methyltransferase (DNMT) activity by upregulating DNMT1 and DNMT3A and selectively promoted regional hypermethylation of specific tumor suppressors 22, 23.

In the current study, we demonstrated that HBx mediated the hypermethylation of the GNA14 promoter and reduced GNA14 expression in HCC. In addition, the downregulation of GNA14 promoted the proliferation and metastasis of HCC.

## Materials and Methods

### Patients and specimens

A total of 50 pairs of human HCC tissues and matched normal tissues were collected from the First Affiliated Hospital of Zhejiang University School of Medicine (Zheyi Hospital). All subjects were obtained from patients who were clinically and histopathologically diagnosed with HCC, who did not receive radiotherapy or chemotherapy treatment before surgery. Tissues were snap-frozen and placed in a -80 °C refrigerator. Written consents were obtained from all patients before participation in the study. The study was approved by the medical ethics committee of the First Affiliated Hospital School of Medicine.

### Cell culture

HCC cell lines (HCCLM3, Huh7, SK-Hep-1, HepG2, Hep3B, PLC/PRF/5, SNU449, Huh6, Li7) and immortalized human hepatocytes (L02) were purchased from the Cell Bank of the Chinese Academy of Sciences (Shanghai, China). HepG2.2.15 cells and immortalized hepatocytes HepLi5 24 were kindly provided by the Infectious Department of the First Affiliated Hospital of Zhejiang University School of Medicine. RPMI-1640 medium (Gibco) with 10% fetal bovine serum (Gibco) was used to culture L02 cells in a 5% CO_2_ incubator (Thermo) at 37 °C. Other cells were cultured in Dulbecco's modified Eagle's medium (Gibco) with 10% fetal bovine serum. G418 was prepared at a concentration of 380 µg/mL to culture HepG2.2.15.

### Receiver operating characteristic curve (ROC) and analysis

ROC curve analysis was applied to evaluate the sensitivity (true positive rate) and specificity (true negative rate) of GNA14 for HCC diagnosis, and we investigated the area under the curve (AUC) using SPSS 26.0.

### Transfection and agents

The transfection method was introduced in our previous study 25. The agents in our study were as follows: small interfering RNAs (siRNAs) targeted to GNA14, DNMT1, DNMT3A, and negative control, which were purchased from RiboBio Co., Ltd. (Guangzhou, China). siRNA targeted to HBx and negative control were purchased from Jima Biotechnology Co., Ltd. (Shanghai, China). HBx overexpressed plasmid, HBVpg3 plasmid (GV146-HBx), were purchased from JiKai Gene Co., Ltd (Shanghai, China). Lentivirus containing the complete open reading frame of GNA14 and GFP (Lentivirus -GFP -GNA14) and plasmid containing GNA14 promoter and luciferase reporter (pGL3-GNA14 Promoter wt-LUC, pGL3-GNA14 Promoter mut-LUC) for the dual luciferase reporter system were purchased from AoQian Co., Ltd (Hangzhou, China). The siRNA sequences are listed in Table S1.

### Cell proliferation assay

Cell proliferation assay was performed using cell counting kit-8 (CCK8, Dojindo, Japan). The method was described in a previous study 25.

### IP or immunoprecipitation coupled with mass spectrometry (IP/MS)

Total proteins were extracted from cells using RIPA (Thermo Fisher Scientific) and 1% protease inhibitor cocktail (Thermo Fisher Scientific). IP was performed according to the manufacturer^'^s instructions (Thermo Fisher Scientific, Cat. No.14321D). Lysates of IP were further verified and analyzed by western blotting and MS.

### Immunofluorescence assay and confocal microscopy

HCC cells were seeded into confocal dishes (20,000 cells/dish). Twenty-four hours after seeding, cells were washed with PBS and fixed with 4% paraformaldehyde for 10 min. The cells were then incubated in 1% Triton X-100 for an hour. Primary antibodies with 8% BSA were added to the dishes, and the cells were incubated for 2 h at room temperature. The cells were incubated with Alexa Fluor 488 and Alexa Fluor 594 labeled IgG (Thermo Fisher Scientific) for 1 h. After washing with PBS, the cells were stained with DAPI (Thermo Fisher Scientific) for 5 min, and imaged with a high-resolution confocal microscope (Olympus, Japan).

### Dual-luciferase reporter system

Cells were seeded in 24-well plates. Twenty-four hours after seeding, cells were transfected with GV146-HBx or GV146-control, or DNMT1 siRNA or DNMT3A siRNA, or negative control. pGL3-GNA14-LUC was further transfected. Dual-luciferase activity was measured using the Luc-Pair ™ Duo-Luciferase HS Assay Kit (Cat. No. LF004) according to manufacturer's instructions.

### *In vivo* tumor model

Subcutaneous tumorigenesis and lung colonization models in nude mice were introduced in a previous study^25^. SK-Hep-1 cells transfected with lentivirus-GFP-GNA14 or lentivirus-control (5 × 10^5^ cells/mL) were injected through the tail vein. Four weeks after injection, nude mice were sacrificed and the lungs were removed and photographed using a small animal three-dimensional imaging system (PERKIN ELMER, IVIS-Sperctrum).

For the nude mouse orthotopic liver tumor model assay, SK- Hep-1 cells transfected with lentivirus-GFP-GNA14 or lentivirus-control (8 × 10^5^ cells/mL) were suspended in medium containing BD Matrigel (BD, 356234) and DMEM medium (1:1), and injected into the livers. All animal studies were designed in accordance with the National Institutes of Health Animal Care and Use Guidelines and approved by the Animal Care Committee of the First Affiliated Hospital of Zhejiang University School of Medicine.

### Demethylation assay

5-Azacytidine (MCE, 320-67-2) was used to demethylate the promoter of GNA14 in HCC cell lines (HCCLM3, Huh7, SK-Hep-1, Hep3B, and HepG2). Cells were seeded into 6-well plates (3 × 10^5^ cells/well). After 24 h of culture, 5-Azacytidine (5 μM) was added to each well. After 48 h of treatment with azacytidine (5 μM), cells were harvested for further analysis.

### 3D cell culture

SK-Hep-1 cells transfected with lentivirus-GFP-GNA14 or lentivirus-control were seeded into the medium (1 × 10^5^ cells/mL) containing BD Matrigel (BD, 356234) and DMEM medium (1:2) in 24-well plates. Seven days after seeding, the 3D spheres of cells were photographed with a fluorescence microscope (OLYMPUS, IX81).

### DNA methylation sequencing analysis

DNA methylation levels in human HCC tissues and HCC cell lines were analyzed by Methyl-target (Genesky Biotechnology Inc., Shanghai, China) sequencing on Illumina MiSeq platform according to the manufacturer's protocols, and NGS-based multiple targeted CpG methylation analysis.

### Bioinformation analysis

The mRNA expression and the methylation level of genes in HCC from the TCGA database were analyzed using R studio (limma package). The methylation levels of the probes in the GNA14 promoter were obtained from Shiny Methylation Analysis Resource Tool (SMART) (http://www.bioinfo-zs.com/smartapp/). The data of GSE94660 was obtained from the GEO dataset (https://www.ncbi.nlm.nih.gov/geo/). The Wurmbach Liver data was obtained from Oncomine (https://www.oncomine.org/resource/login.html).

### Colony formation assay

Cells transfected with lentivirus-GFP-GNA14 or lentivirus-control were seeded in 6-well plates (500 cells/well). After culturing for 2 weeks in a 5% CO_2_ incubator (Thermo) at 37 °C, cells were stained with jimosa (Nanjing, Jiancheng Technology). Each well of the plate was imaged and analyzed.

### Transwell assay

Migration and Matrigel-Invasion Assays were performed to assess the HCC cell metastasis. A total of 50,000 cells transfected with lentivirus-GFP-GNA14 or lentivirus-control were seeded in the upper chamber containing 200 µL FBS-free medium. Medium (500 µL) containing 10% FBS was added in the lower chamber. After culture for 24 h or 48 h, the upper chamber was removed and stained with jimosa (Nanjing, Jiancheng Technology). Finally, the cells on the lower membrane were imaged and analyzed. In Matrigel-Invasion Assays, Matrigel (Corning, Cat #356231) was added to the upper chamber and placed in an incubator at 37 °C for 30 min before cell seeding.

### Cell proliferation assay

Cells transfected with lentivirus-GFP-GNA14 or lentivirus-control were seeded in a 96-well plate (4000 cells/well). The CCK-8 assay (MCE, Cat#HY-K0301) was performed according to manufacturer's instructions.

### RNA extraction and qRT-PCR

Total RNA was extracted according to the manufacturer's instructions (ES Science, Cat#RN001). qRT-PCR was performed as previously described 25. The primers used are listed in Table S2.

### Protein extraction and western blotting

Total protein was extracted with RIPA containing 1% protease inhibitors (Thermo Fisher, Cat#A32965). The BCA assay was performed to test the protein concentration. Western blotting was performed as previously described 25. The antibodies are listed in Table S3.

### Cycloheximide (CHX) treatment

Cells transfected with lentivirus-GFP-GNA14 or lentivirus-control were seeded in 6-well plates (3*10^5^ cells/well) and CHX (10uM) was added in each well. After treated for 0, 1, 2, 3, 4, 5, 6, 8,12 hours, cells were collected for Western Blot.

### Chromatin Immunoprecipitation

Cells were cultured in a 15 cm dish. When confluency reached approximately 90%, cells were harvested for chromatin immunoprecipitation (CHIP) according to the manufacturer's instructions (CST, Cat#9003s).

### Immunohistochemistry (IHC) staining

Tissues were fixed with 4% formaldehyde. After embedding in paraffin, tissues were sliced into 4 μm-thick slices and placed in an oven at 65 °C overnight. Different gradient alcohol concentrations were used to re-hydrate cells in tissues. After retrieving the antigen and quenching the endogenous peroxidase activity, slices were incubated with antibodies in PBS with 5% FBS overnight. After incubation with the secondary antibody, slices were stained with the DAB (Origene, Cat#IB000103). Images were captured.

The antibodies are listed in Table S3.

### Statistical analysis

Data from this study were expressed as mean ± standard deviation (SD). SPSS 26.0 was used for the statistical analyses in our study. Differences between experimental groups were compared using Student's t-test. Survival analysis was calculated using the Kaplan-Meier method. Pearson analysis was used to analyze the correlation between two groups. Statistical significance was indicated by a p-value < 0.05. *p < 0.05. **p < 0.01. ***p < 0.001. ****p < 0.0001.

## Results

### GNA14 is identified as a potential methylation-related tumor suppressor of HCC after data integration

**Figure 1:**
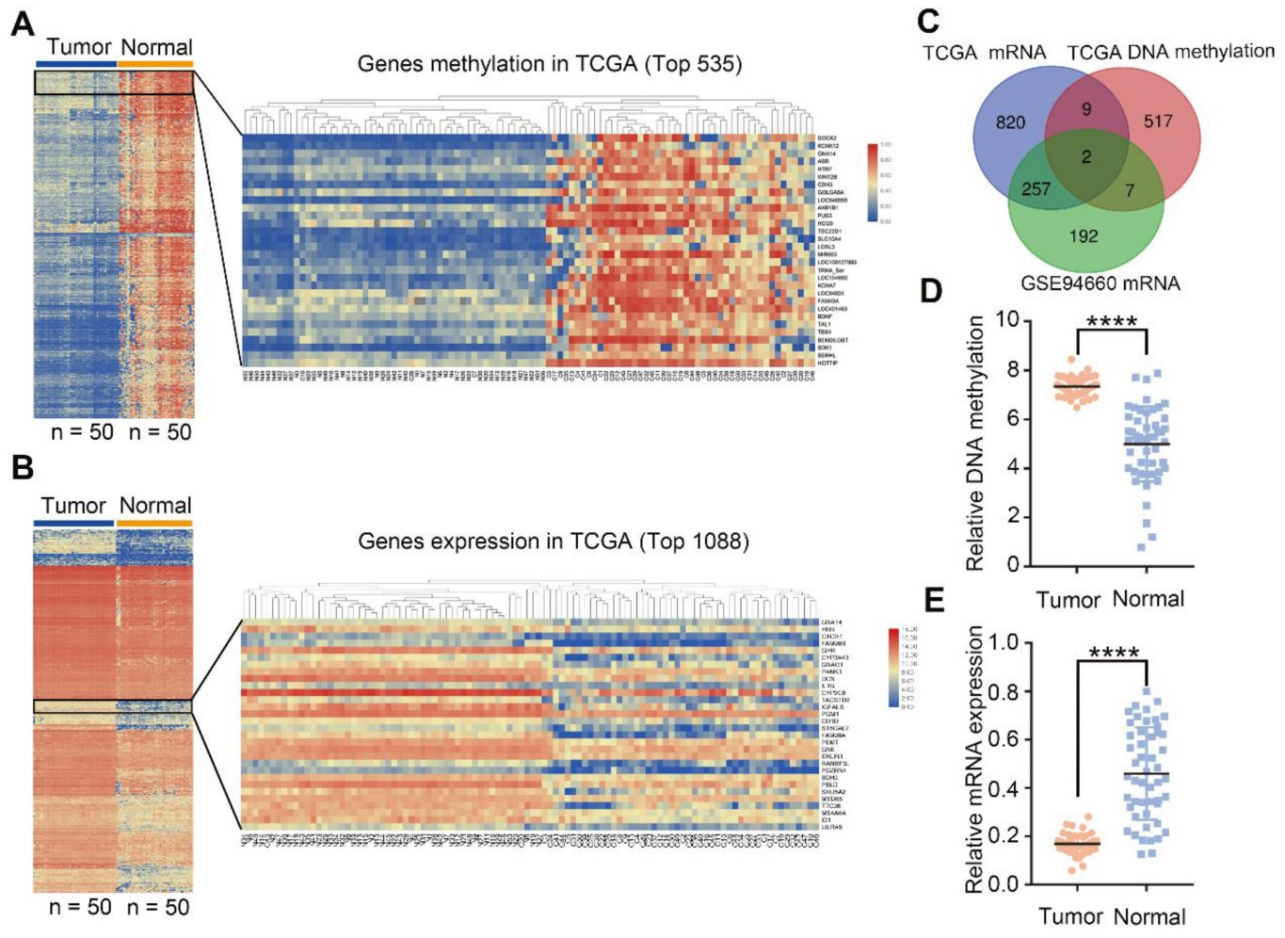
GNA14 is identified as a potential methylation-related tumor suppressor of HCC after data integration. (A) Heatmap analysis of differential DNA methylation expression profiles between normal tissues and cancer tissues from TCGA database (logFC >1, FDR <0.0001). (B) Heatmap analysis of differential mRNA expression profiles between normal tissues and cancer tissues from TCGA database (logFC<-1, FDR<0.001). (C) Venn diagram of the gene screening. (D) GNA14 total DNA methylation of matched tumor tissues and normal tissues in TCGA. (E) GNA14 mRNA level of matched tumor tissues and normal tissues in TCGA. *p < 0.05. **p < 0.01. ***p < 0.001.

To obtain the most important potential tumor suppressors in HCC, we analyzed the mRNA expression in TCGA and GSE94660, and analyzed DNA methylation in TCGA. From the TCGA-methylation database, we obtained 535 differentially expressed genes (DEGs), whose methylation level in HCC tissues was the most significant (Figure 1A; logFC > 1, FDR < 0.0001). From the TCGA mRNA database, we obtained 1,088 DEGs with the most significantly downregulated levels in tumor tissues compared with normal matched tissues (Figure 1B; logFC < -1, FDR< 0.001). Moreover, 458 significantly down-regulated genes (green dots) were ascertained in GSE94660 (Figure S1A, left panel; logFC < -1, FDR< 0.05). Finally, two overlapping genes (GNA14 and TBX15) from the above-mentioned three datasets were obtained (Figure 1C). Except for the significant upregulation of DNA methylation (Figure 1D) and downregulation of mRNA level (Figure 1E); GNA14 was negatively correlated with the prognosis of HCC patients, while TBX15 was not (Figure S1B). Therefore, we aimed to investigate the role of GNA14 in HCC.

### Decreased expression of GNA14 in HCC

**Figure 2:**
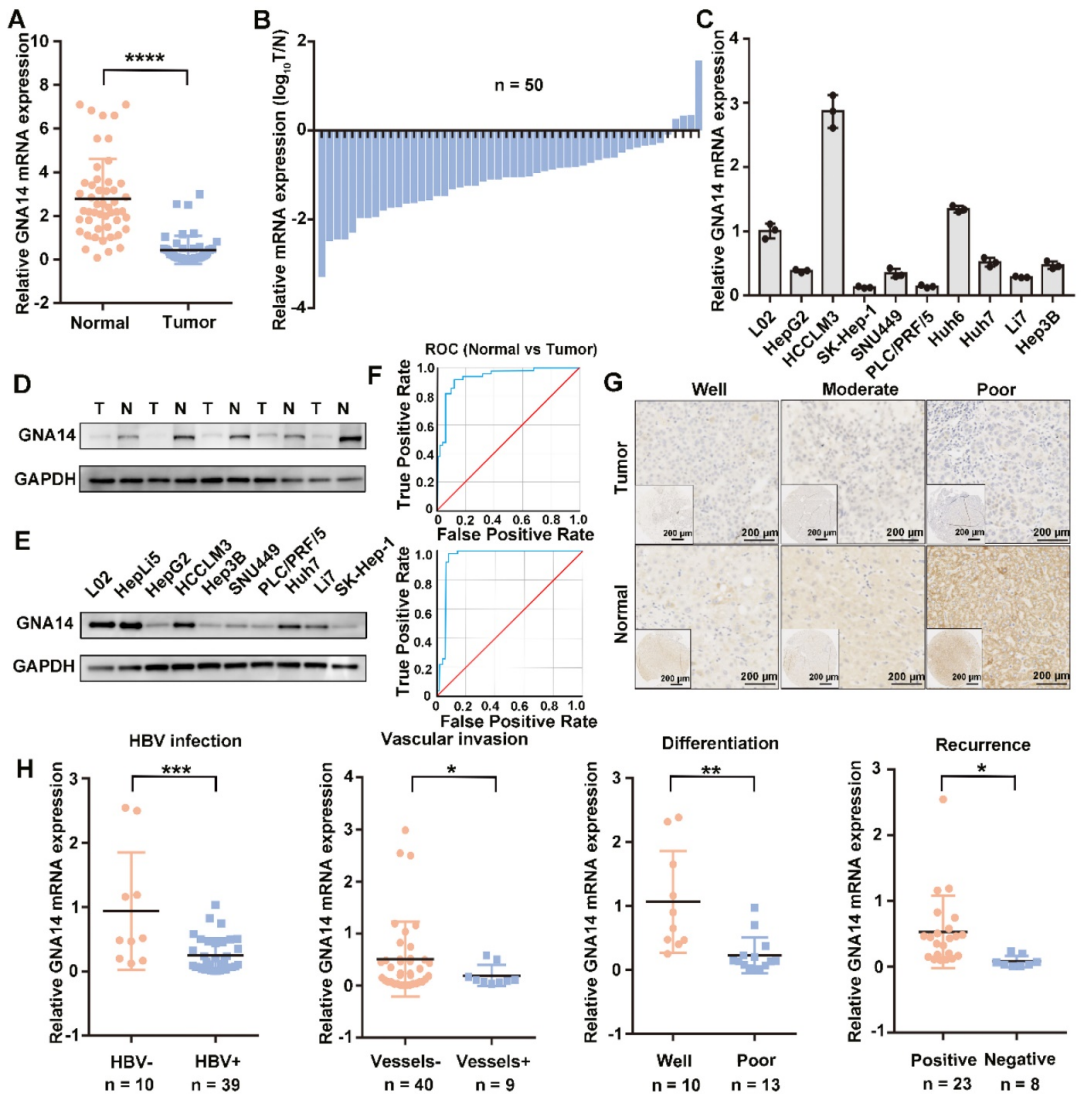
GNA14 is downregulated in HCC. (A) qRT-PCR analysis of GNA14 mRNA level in tumor tissues and matched normal tissues from our hospital. (B) GNA14 mRNA level shown in log_10_(tumor/normal) form. (C) qRT-PCR analysis of GNA14 mRNA levels in HCC cell lines and L02 cells. (D, E) Western blot analysis of GNA14 protein levels in matched tumor tissues and normal tissues from our hospital and protein levels in HCC cell lines, L02 and HepLi5 cells. (F) ROC (receiver operating characteristic) curve in normal tissues and tumor tissues respectively in TCGA (AUC=0.948) and our hospital (AUC=0.935). (G) IHC staining analysis of GNA14 protein in matched tumor tissues and normal tissues. The expression levels of GNA14 in tumor tissues at different levels of differentiation (well-differentiated, moderate-differentiated, and poor-differentiated) are shown. (H) Relationship between GNA14 and the clinical characteristics. *p < 0.05, **p < 0.01, ***p < 0.001, ****p < 0.0001. Statistical significance was determined by unpaired t-test.

To verify the expression of GNA14 in HCC, we initially used qRT-PCR to detect the mRNA expression of GNA14 in tumor tissues (n = 50) and matched adjacent normal tissues (n = 50) from the First Affiliated Hospital of Zhejiang University School of Medicine. In 46 pairs (92%), the mRNA expression of GNA14 mRNA in tumor tissues was downregulated (7.2-fold) compared to that in matched normal tissues (Figure 2A-B). In addition, seven HCC cell lines showed lower GNA14 mRNA expression compared with L02 cell lines, except for HCCLM3 and Huh6 (Figure 2C). The protein levels of several paired samples and cell lines were verified (Figure 2D-E, Figure S1C-D). We conducted receiver operating characteristic (ROC) analysis to evaluate the sensitivity and specificity of GNA14 for the diagnosis of HCC in TCGA database (AUC = 0.948) and HCC tissues from our hospital (AUC = 0.935) (Fig. 2F). Immunohistochemistry (IHC) revealed that GNA14 expression was decreased in the tumor tissues compared with the matched normal tissues (Figure 2G). We verified in the GSE94660 cohort that GNA14 expression was lower in tumors (Figure S1A, right panel). We also verified in the Oncomine database (https://www.oncomine.org/) that the Wurmbach cohort also had differences in GNA14 expression (Figure S1E). Moreover, GNA14 expression was associated with clinical characteristics. In the HCC samples from our hospital, the mRNA expression of GNA14 was downregulated in HBV-infected tissues (4.55-fold) and vascular invasion tissues (2.63-fold). GNA14 expression was also decreased in poorly differentiated tissues (4.6-fold) and tissues from recurrent patients (6.47-fold) (Figure 2H). In the Wurmbach cohort, GNA14 expression was negatively correlated with tumor size and tumor grade (Figure S1E). In TCGA, the mRNA expression of GNA14 was negatively correlated with vascular invasion (Figure S1F).

### Methylation of the GNA14 promoter is upregulated in HCC

**Figure 3:**
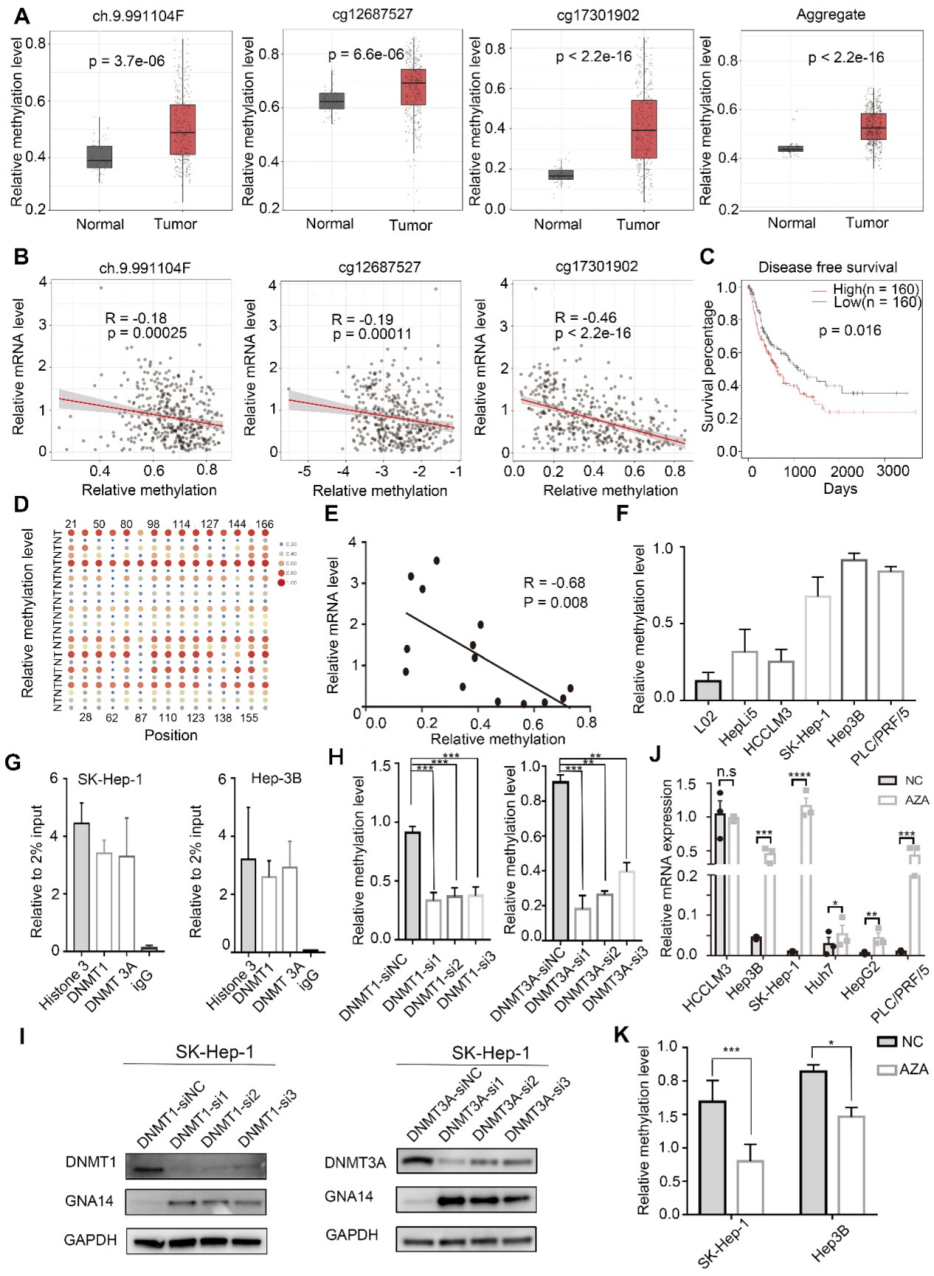
Hypermethylation of GNA14 leads to downregulated expression in HCC. (A) Relative methylation level of three GNA14 probes (ch.9.991104F, cg12687527, cg17301902) in HCC patients in TCGA. (B) Pearson's correlation between methylation level and mRNA level of GNA14 in TCGA. (C) Kaplan-Meier survival analysis based on high or low GNA14 methylation. (D) The heatmap of the methyl target sequencing results indicated the methylation levels of 15 methylation sites in the target CpG islands (Genomic position 1: 80262823-80262968) of the GNA14 promoter region in 12 pairs of matched tumor tissues and normal tissues in our hospital. “T” refers to tumor tissue and “N” refers to normal tissue. The horizontal axis was the probe sites, and the number represents the location of this site on the target CpG island. For example, ”22” represents the 22th base site on CpG island. Each point represents the degree of DNA methylation of the tissue. The larger the size of the dot, the redder the color, indicating the higher the degree of methylation of the tissue. (E) Pearson's correlation between methylation level and mRNA level of GNA14 in different HCC tissues from our hospital. (F) Methyl-target sequencing results showing GNA14 promoter methylation level in HCC cell lines, L02 and HepLi5 cells. (G) ChIP-qPCR analysis showing the binding of DNMT1 and DNMT3A to the GNA14 promoter region in SK-Hep-1 and Hep3B. Histone 3 was positive control group. IgG was negative control group. (H) DNA methylation sequencing of SK-Hep-1 knocking down DNMT1 or DNMT3A. (I) Western blot analysis of GNA14 protein level after DNMTs (DNMT1 or DNMT3A) knockdown in SK-Hep-1. (J) qRT-PCR analysis of GNA14 mRNA level in HCC cell lines after 5-Azacytidine treatment. (K) DNA methylation sequencing of SK-Hep-1 and Hep3B cells treated with 5-Azacytidine. *p < 0.05. **p < 0.01. ***p < 0.001. ****p < 0.0001. Statistical significance was determined by unpaired t-test. Kaplan-Meier survival analysis was performed to analyze the survival percentage. Pearson analysis was used to analyze the correlation between two groups.

To explore the methylation status of GNA14 in TCGA, the Shiny Methylation Analysis Resource Tool (SMART) was used for analysis. We found that three probes of GNA14 (ch9.991104F, cg12687527, and cg17301902) showed upregulation of methylation in tumor tissues compared to normal tissues (Figure 3A,). In addition, a negative correlation between the methylation level of GNA14 and the mRNA level was also found in the TCGA database (Figure 3B). Moreover, we confirmed that upregulation of GNA14 methylation was associated with poor prognosis in TCGA (*p =* 0.016) (Figure 3C).

With the help of METHPRIMER (http://www.urogene.org/cgi-bin/methprimer/methprimer.cgi), we analyzed the promoter region of GNA14 and found two obvious CpG islands (Genomic position 1: 80262823-80262968; Genomic position 2: 80263568-80263728) (Figure S2A). Then, the methylation levels of these two CpG islands were detected in 12 pairs of HCC and matched adjacent normal tissues. Through DNA methylation sequencing, we found that one of the CpG islands (genomic position: 80262823-80262968) was hypermethylated in the tumor (Figure 3D), while the other one was not (Figure S2B). The mRNA levels of tissue samples used for DNA methylation sequencing were also tested. The results revealed that the mRNA expression of GNA14 was downregulated in tumor tissues (Figure S2C), and GNA14 mRNA expression was significantly negatively correlated with the methylation level of the DNA methylation sequencing region (R = -0.68, *P =* 0.008) (Figure 3E). HCC cell lines (SK-Hep-1, HCCLM3, Hep3B and PLC/PRF/5) and normal liver cell lines (L02, HepLi5) were also tested for methylation levels. Methylation sequencing results indicated that the methylation status of HCC cell lines was upregulated compared with normal liver cell lines, especially in SK-Hep-1, Hep-3B, and PLC/PRF/5 (Figure 3F). ChIP-qPCR analysis demonstrated the binding of DNMT1 and DNMT3A to the GNA14 promoter region in SK-Hep-1 and Hep3B cells (Figure 3G). When DNMT1 and DNMT3A were knocked down in SK-Hep-1 cells, methylation sequencing showed that the methylation level of CpG islands was downregulated (Figure 3H), while GNA14 protein expression increased (Figure 3I, Figure S2D). After 48h of HCC cell line stimulation with the DNA methylase inhibitor 5-azacytidine (SK-Hep-1, PLC/PRF/5, Hep3B, HCCLM3, HepG2 and Huh7), the mRNA level of GNA14 increased significantly except for HCCLM3 (Figure 3J). The results of DNA methylation sequencing revealed that the DNA methylation levels of HCC cell lines (SK-Hep-1, Hep3B) treated with 5-azacytidine were significantly downregulated (Figure 3K). In summary, we thought that the downregulation of GNA14 expression in tumors was probably caused by the upregulation of DNA methylation level in its promoter region.

### Hypermethylation of the GNA14 promoter is caused by HBx

**Figure 4:**
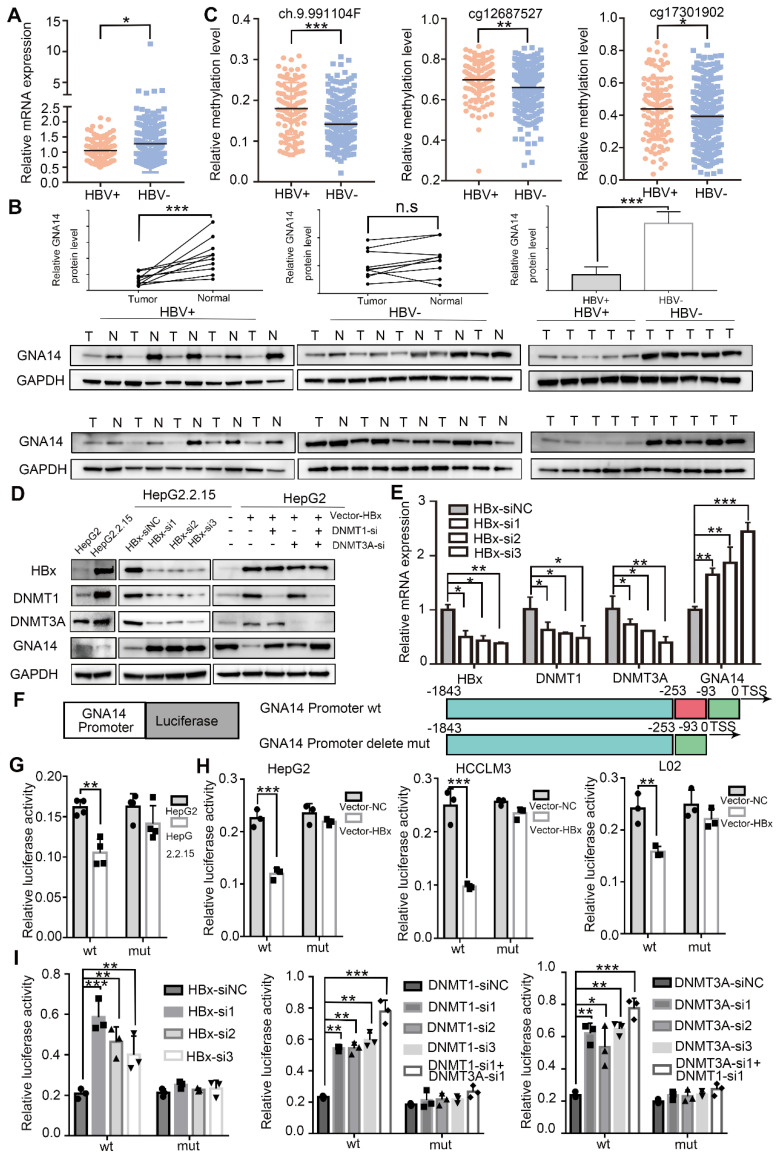
HBx causes hypermethylation of GNA14 promoter. (A) GNA14 mRNA level of HCC patients with or without HBV infection in TCGA. (B) Western blot analysis of GNA14 protein levels in moderately differentiated HCC tissues with or without HBV infection in our hospital. (C) GNA14 methylation level of HCC tissues in three probes (ch.9.991104F, cg12687527, cg17301902) with or without HBV infection in TCGA. (D) Western blot analysis of HBx, DNMTs, and GNA14 protein levels in HepG2 and HepG2.2.15 cells. Western blot analysis of HBx, DNMTs and GNA14 protein levels after HBx knockdown (HBx-si or HBx-si-NC) in HepG2.2.15 cells or HBx overexpression (vector-NC or vector-HBx) in HepG2 cells, or combining HBx overexpression and DNMTs (DNMT1 and DNMT3A) knockdown in HepG2 cells. (E) Relative mRNA expression of HBx, DNMTs and GNA14 after HBx knockdown (HBx-si or HBx-si-NC) in HepG2.2.15 cells. (F) Construction of wild-type GNA14 promoter luciferase reporter gene system and deletion mutant GNA14 promoter luciferase reporter gene system. (G) Relative luciferase activity of GNA14 promoter and mutant GNA14 promoter in HepG2 and HepG2.2.15 cells. (H) Relative luciferase activity of GNA14 promoter and mutant GNA14 promoter in HBx overexpressed (Vector-NC or Vector-HBx) HepG2, HCCLM3 and L02 cells. (I) Relative luciferase activity of GNA14 promoter and mutant GNA14 promoter after HBx knockdown (HBx-si-NC or HBx-si) or DNMTs (DNMT1 and DNMT3A) knockdown in HepG2.2.15 cells. *p < 0.05, **p < 0.01, ***p < 0.001, n.s.: no significance. Statistical significance was determined by unpaired t-test.

According to the TCGA database, patients infected with HBV showed lower GNA14 mRNA levels (Figure 4A). Hence, the GNA14 protein in tumor tissues of patients with (n = 10) or without (n = 10) HBV infection was measured, which revealed that patients with HBV infection had lower GNA14 levels. And HBV positive HCC tissues showed lower GNA14 level than normal tissue while HBV negative group was not significant (Figure 4B). In addition, the methylation level of the GNA14 promoter (ch9.991104F, cg12687527, cg17301902) was related to HBV infection in the TCGA methylation database by SMART (Figure 4C). Our DNA methylation sequencing results showed that HBV-positive samples had higher GNA14 DNA methylation levels than HBV-negative samples (Figure S2E).

HepG2.2.15 was an HCC cell line derived from HepG2 infected with HBV genomic DNA 26. Compared with HepG2, the protein and mRNA expression of DNMT1, and DNMT3A in HepG2.2.15 increased significantly, while GNA14 decreased (Figure 4D, Figure S2F). DNA methylation sequencing results suggested that the promoter CpG island methylation level of HepG2.2.15 cells were higher than that of HepG2 (Figure S2G).

HBx has been shown to regulate DNA methylation by affecting DNMTs. We used Co-IP to verify the relationship between HBx and DNMT, and confirmed that HBx physically interacted with DNMT1 and DNMT3A. And there was no interaction between HBx and GNA14. (Figure S2H). Knockdown of HBx reduced the expression of DNMT1 and DNMT3A, but upregulated the expression of GNA14 in HepG2.2.15 cells (Figure 4D-E). Furthermore, HBx-overexpressed plasmid was transfected into HepG2 cells, upregulation of DNMT1 and DNMT3A, and downregulation of GNA14 in the HBx overexpression group was observed (Figure 4D). After transfection of the HBx overexpression plasmid, knockdown of DNMT1 or DNMT3A by RNA interference rescued the expression of GNA14. Knockdown of DNMT1 and DNMT3A at the same time significantly increased the expression of GNA14 (Figure 4D).

The dual luciferase reporter system further explored the changes in the activity of the GNA14 promoter in HepG2, HepG2.2.15, HCCLM3, and L02 cells (Figure 4F). In addition to the wild-type GNA14 promoter luciferase reporter system (GNA14 Promoter wt), we also constructed a mutant GNA14 promoter luciferase reporter system (GNA14 Promoter Delete mut) with a deletion of the target methylation site (genomic position: 80262823-80262968) (Figure 4F). In the wt group, compared with HepG2 cells, the promoter activity in HepG2.2.15 cells was significantly down-regulated (Figure 4G, left panel). But when the target methylation site encountered a deletion mutation, the difference between the two cell lines disappeared (Figure 4G, right panel). When HBx was overexpressed in HepG2, HCCLM3 and L02, the activity of the GNA14 promoter was down-regulated, but when the target methylation site was mutated, the activity could be restored (Figure 4H). We also knocked down HBx, DNMT1 or DNMT3A in HepG2.2.15 cells by siRNA. The activity of the GNA14 promoter was significantly increased in the respective knockdown groups. However, when the deletion mutation occurred at the target DNA methylation site, the activity change of the GNA14 promoter was eliminated (Figure 4I).

Through DNA methylation sequencing, we detected changes in the methylation level of HCC cell lines overexpressing HBx (L02, and HepLi5) and found that the HBx overexpression group showed significant hypermethylation (Figure S2I).

Taken together, these results indicated that HBx could affect the DNA methylation of the GNA14 promoter by regulating DNMT1 and DNMT3A, leading to the downregulation of GNA14 expression.

### GNA14 suppresses the proliferation of HCC by promoting Notch1 cleavage

**Figure 5:**
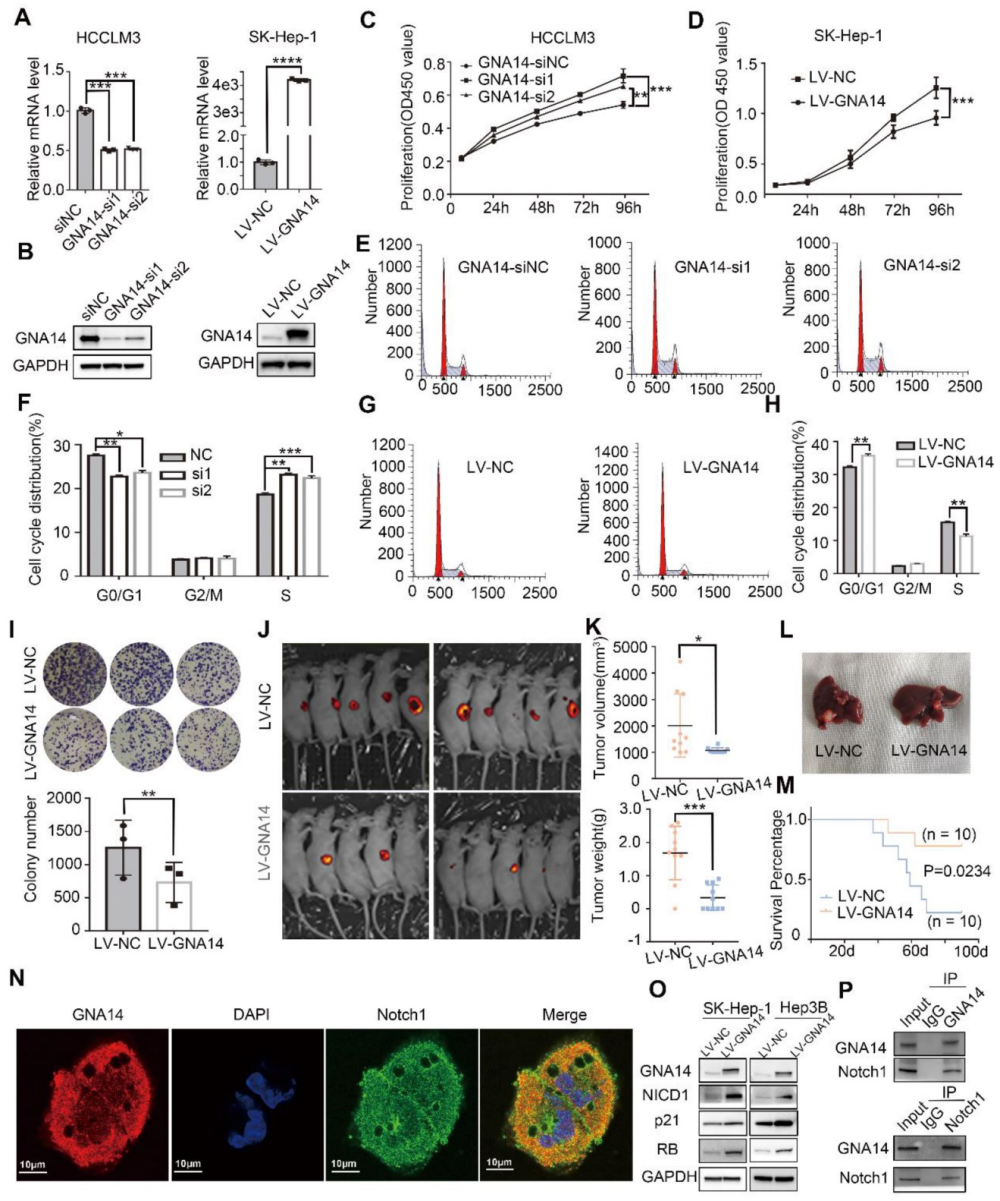
GNA14 suppresses the proliferation of HCC by promoting Notch1 cleavage. (A) qRT-PCR was performed to verify the effect of GNA14 knockdown and overexpression. (B) Western blot was performed to verify the effect of GNA14 knockdown in HCCLM3 cells and overexpression in SK-Hep-1 cells. (C) CCK8 assay was performed to determine the proliferation of HCCLM3 cells infected with GNA14 siRNA or si-negative control (si-NC). (D) CCK8 assay was performed to determine the proliferation of SK-Hep-1 cells infected with GNA14 lentivirus (LV-GNA14) or lentivirus negative control (LV-NC). (E, F) Effects of GNA14 knockdown on the cell cycle distribution of HCCLM3 cells. (G, H) Effects of GNA14 overexpression on the cell cycle distribution of SK-Hep-1 cells. (I) Colony assay with cells with LV-GNA14 or LV-NC. (J) Fluorescent image of subcutaneous human HCC xenograft model with GNA14 stably overexpressed cells or control cells. (K) Tumor volume and weight of HCC xenograft were measured. (L) Orthotopic liver tumor model in nude mice with GNA14 stably overexpressed cells or control cells. (M) The survival of orthotopic tumor model mice was analyzed by Kaplan-Meier analysis. (N) Immunofluorescence analysis of cytoplasmic distribution of GNA14 and Notch1 in SK-Hep-1 cells. (O) The expression level of NICD1, RB, and P21 in GNA14 overexpressed SK-Hep-1 and Hep3B cells was examined by western blot. (P) Lysates of SK-Hep-1 cells were immunoprecipitated with anti-GNA14 or anti-Notch antibody, and were examined using western blot analysis. *p < 0.05, **p < 0.01, ***p < 0.001, ****p < 0.0001. Statistical significance was determined by unpaired t-test. Kaplan-Meier analysis was performed to analyze the survival percentage.

To explore whether GNA14 affects HCC function, we used lentivirus to stably overexpress GNA14 in SK-Hep-1 and Hep3B cells with low GNA14 expression, and siRNA to inhibit GNA14 in HCCLM3 and Huh7 cells with high GNA14 expression (Figure 5A-B and Figure S3A). The CCK8 assay showed that knockdown of GNA14 improved cell viability (Figure 5C and Figure S3B), while over-expression of GNA14 suppressed cell viability (Figure 5D and Figure S3C). Moreover, we quantified cell cycle distribution using flow cytometry and found that knocking down the expression of GNA14 decreased the percentage of G0/G1 phase and increased the S phase (Fig. 5E-F), while overexpression of GNA14 increased the percentage of G0/G1 phase and decreased the S phase (Figure 5G-H). In addition, a colony formation assay showed that overexpression of GNA14 reduced the number of clones, while knocking down GNA14 could increase the number of clones. (Figure 5I and Figure S3D). Annexin V and propidium iodide staining were used to evaluate whether GNA14 affected HCC cell apoptosis. It was found that the GNA14 overexpression group did not show a significant pro-apoptotic phenotype (Figure S3E). We cultured SK-Hep-1 cells in a three-dimensional culture method and found that the cell cluster from GNA14 overexpression group had smaller diameter and a smoother appearance than the control group (Figure S3F).

To investigate the role of GNA14 in tumor growth *in vivo*, GNA14 overexpressed SK-Hep-1 cells together with negative control cells were injected subcutaneously into nude mice for growth analysis. As a result, the growth of the subcutaneous human liver cancer xenograft in nude mice of the GNA14 overexpression group was inhibited (Figure 5J). Meanwhile, the tumor volume and weight also decreased (Figure 5K, and Figure S4A). To further investigate the suppressive role of GNA14, GNA14 overexpressed and negative control SK-Hep-1 cells were also used to establish an orthotopic liver tumor model in nude mice. Four weeks after surgery, positron emission tomography-computed tomography (PET-CT) results confirmed the growth of tumors in the livers of mice (Figure 5L and Figure S4B). We recorded the survival status of nude mice; survival analysis demonstrated that nude mice in the GNA14 overexpression group had a better prognosis (Figure 5M). Several potential interacting proteins were screened by immunoprecipitation coupled with mass spectrometry (IP/MS), which includes Notch1 (Figure. S4C). Notch1 is a subtype of Notch pathway receptor 27 and activated by intracellular cleavage by γ-secretase to produce an active Notch intracellular domain (NICD), which was reported to inhibit HCC proliferation by promoting cyclin p21 28. Through immunofluorescence, we initially observed the co-localization of GNA14 and Notch1 in SK-Hep-1 cells (Figure 5N).

The expression relationship between GNA14, the Notch pathway, and the RB pathway was further analyzed. When GNA14 was overexpressed in SK-Hep-1, the expression of active Notch1 intracellular domain (NICD1) increased, and the expression of cyclin p21 and RB also increased (Figure 5O). The IHC staining and western blot results of subcutaneous tumor tissues of nude mice also suggested that the expression of NICD1 was significantly increased in the GNA14 overexpression group (Figure S4D). Immunoprecipitation further verified the interaction between GNA14 and Notch1 (Figure 5P). After overexpression of GNA14, we stimulated SK-Hep-1 cells with 10 mM Tangeretin (Notch1 inhibitors). CCK8 and colony formation assays revealed that Tangeretin can reverse the inhibitory effect of GNA14 (Figure S4E). These results suggested that GNA14 inhibited HCC proliferation by promoting the cleavage of Notch1.

### GNA14 suppresses the metastasis of HCC by inhibiting JMJD6

**Figure 6:**
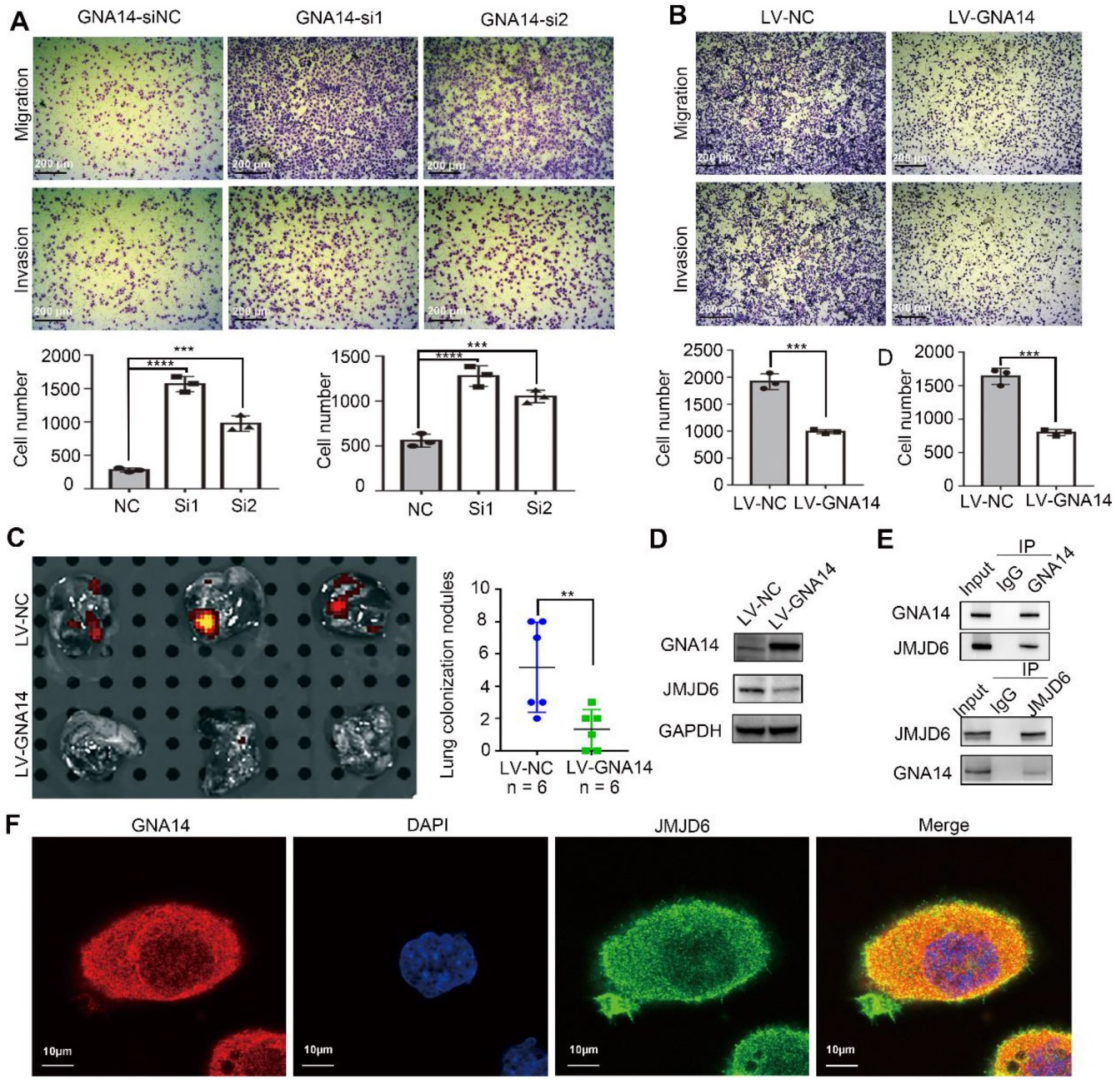
GNA14 suppresses HCC metastasis by inhibiting JMJD6. (A) The effect of knockdown of GNA14 in HCCLM3 on cell migration and invasion was detected by transwell assay. (B) The transwell assay was used to detect the effect of overexpression of GNA14 in SK-Hep-1 on cell migration and invasion. (C) Tumor model of lung colonization in nude mice with GNA14 stably overexpressed cells or control cells. Photon flux detection was performed to show lung metastasis nodules, which were then counted. (D) The expression level of JMJD6 in GNA14 overexpressed SK-Hep-1 cells was examined by western blot analysis. (E) Lysates of SK-Hep-1 cells were immunoprecipitated with anti-GNA14 or anti-JMJD6 antibody, and were examined by western blot analysis. (F) Immunofluorescence analysis of the cytoplasmic distribution of GNA14 and JMJD6 in SK-Hep-1 cells. *p < 0.05, **p < 0.01, ***p < 0.001, ****p < 0.0001. Statistical significance was determined by unpaired t-test.

In addition to proliferation, metastasis is also an important feature of tumor cells. We previously also found that the expression of GNA14 was negatively correlated with vascular invasion in HCC patients (Figure 2B). Several assays were performed to evaluate the effect of GNA14 on tumor metastasis. A transwell assay was used to test the effect of GNA14 on the migration and invasion of HCC cells. The results showed that knocking down GNA14 could improve the migration and invasion of HCCLM3 and Huh7 cells (Figure 6A, Figure S5A), while overexpression of GNA14 suppressed the migration and invasion of SK-Hep-1 and Hep3B cells (Figure 6B, Figure S5A).

To investigate the role of GNA14 in metastasis *in vivo*, we established a model of lung colonization in nude mice by injecting SK-Hep-1 cells with or without GNA14 overexpression into the tail vein. Based on photon flux detection and histological analysis of lung tissue, we found that GNA14 overexpression was associated with reduced HCC metastasis (Figure 6C and Figure S5B). According to the GCBI analysis (Figure S5C; https://www.gcbi.com.cn/gclib/html/index), Jumonji domain-containing 6 (JMJD6), a member of the Jumonji C domain-containing family of proteins, was considered to be a protein that interacted with GNA14. Previous studies have demonstrated that JMJD6 is an important protein that promotes cell metastasis in many tumors, including HCC 29.

When we overexpressed GNA14 in SK-Hep-1 cells, the expression of JMJD6 significantly decreased (Figure 6D). The immunohistochemical staining and western blot results of subcutaneous tumor tissues of nude mice also indicated that the expression of JMJD6 was significantly increased in the GNA14 overexpression group (Figure S4D). Meanwhile, Co-IP further confirmed that GNA14 interacted with JMJD6 (Figure 6E). In addition, the immunofluorescence results demonstrated that GNA14 and JMJD6 co-localized in the cytoplasm and membrane of HCC cells (Figure 6F). Furthermore, mass spectrometry identification indicated that JMJD6 was the protein captured by GNA14 (Figure S5D). We found that over-expression of GNA14 cannot affect the transcription of JMJD6 (Figure S5E). We also found that when SK-Hep-1 cells were treated with 10ug/ml cycloheximide (CHX), the degradation rate of JMJD6 in the GNA14 overexpression group was faster (Figure S5F). When the JMJD6 plasmid was transferred into SK-Hep-1 and Hep3B cells overexpressing GNA14, the inhibitory effect of GNA14 on invasion and migration was reversed (Figure S5G). Therefore, these results indicated that GNA14 may inhibit the metastasis of HCC by inhibiting the expression of JMJD6.

## Discussion

Despite the rapid development of diagnosis and treatment, HCC is still considered one of the most lethal tumors. Therefore, it is of great significance to further reveal the mechanism of liver cancer, and to develop more scientific and effective biomarkers and treatment strategies.

Heterotrimeric G protein complex is a type of intracellular guanine nucleotide binding protein composed of Gα, Gβ, and Gγ subunits, which can mediate G protein coupled receptor (GPCR) signal transduction 30. There are 16 Gα subunit proteins. According to their protein sequences and functions, they can be divided into four families: G_s_, G_i/o_, G_q/11_, and G_12/13_ 31. Different Ga protein families have different functions in the occurrence and development of tumors, such as GNA12 and GNA13, which belong to the GNA12/13 family and play a role in promoting oncogenic transformation and tumor cell growth 5. GNAi3, belonging to the G_i/o_ family, can inhibit the migration and invasion of HCC cells 6. Uveal melanoma is usually caused by mutations in GNAQ or GNA11, which belong to the G_q/11_ family 32. GNA14 is also a member of the G_q/11_ family, and its mechanism in the occurrence and development of HCC is unknown.

Through combined analysis of TCGA and GEO databases, a potential methylation-related tumor suppressor, GNA14, was found. GNA14 has been reported to inhibit the proliferation of various tumors 4, 33. We initially found that the mRNA and protein levels of GNA14 were significantly decreased in HCC tissues compared with the matched adjacent tissues. It is worth noting that the expression of GNA14 was significantly negatively correlated with HBV infection, vascular invasion, tumor differentiation, and prognosis. According to the Oncomine database, GNA14 expression was downregulated in tumors and inversely related to tumor size and tumor grade. This evidence suggests that GNA14 has the potential to inhibit the occurrence and development of HCC.

DNA methylation plays an important role in tumorigenesis 17, 34. By up-regulating or down-regulating the DNA methylation level of the target gene, the expression of tumor suppressors or oncogenes can be regulated, thereby affecting the occurrence and development of tumors 35. In the TCGA database, there was a hypermethylation state in the promoter region of the GNA14 gene in liver cancer tissues. DNA methylation levels were negatively correlated with GNA14 mRNA levels. Using DNA methylation sequencing, we detected the methylation status of the obvious CpG island in the promoter region of GNA14. The results showed that the detected region had a hypermethylation status and was also inversely related to GNA14 mRNA levels. Meanwhile, 5-Azacytidine stimulation or knockdown of the expression of DNA methylases downregulated the methylation level of target CpG island and increased the expression of GNA14. Hence, it was demonstrated that GNA14 was indeed regulated by DNA methylation.

HBV infection is an important cause of HCC. HBV promotes the development of HCC through a series of complex mechanisms. HBV DNA can integrate into the host genome and directly induce chromosomal alterations, genomic instability, and insertion mutagenesis of various cancer-related genes. In addition, HBV can indirectly promote the occurrence of HCC by affecting cell functions such as cell cycle, oxidative stress, telomere maintenance, and epigenetic plasticity 36. The expression of HBV proteins (especially HBx) has been reported to induce abnormal histone acetylation and DNA methylation, promoting the activation of oncogenes and silencing of tumor suppressors. HBx can directly promote the activity of DNA methylase in a combined manner, and can also indirectly promote the upregulation of DNA methylase by inhibiting microRNAs 37-39. Since we found that the expression of GNA14 was significantly correlated with HBV infection, we suspected that HBV infection was a potential cause of DNA methylation change in GNA14. In the TCGA methylation database, GNA14 methylation levels were associated with HBV infection, which was consistent with the results of methylation sequencing of our HCC samples. Compared with HepG2 cells without HBV, HepG2.2.15 cells carrying HBV DNA expressed higher levels of HBx, but lower GNA14. And the DNA methylation level of target CpG island in HepG2.2.15 was higher than HepG2. When suppressing the expression of HBx in HepG2.2.15, the expression of DNMT1 and DNMT3A decreased, and GNA14 increased. Overexpression of HBx in HepG2 cells increased the expression of DNMT1 and DNMT3A, while GNA14 decreased. In addition, knockdown of DNMT1 and DNMT3A on the basis of overexpression of HBx rescued GNA14 expression. The interaction between HBx and DNMT has also been confirmed. After the HBx plasmid was transfected into L02 and HepLi5 cells, the methylation level of the target CpG island was significantly upregulated. These results suggested that HBx was related to DNA methylation and GNA14. Similarly, the dual luciferase reporter assay revealed that HBx promoted the hypermethylation of the GNA14 promoter by enhancing the expression of DNA methylase, thereby inhibiting the expression of GNA14.

As a potential tumor suppressor, whether GNA14 can inhibit the function of HCC is unknown. Rapid proliferation is a major feature of tumors 40, 41. The CCK8 assay showed that GNA14 could inhibit tumor cell proliferation in HCC by inducing G0 / G1 phase retardation in the HCC cell cycle. The results of *in vivo* experiments showed that the mice in the GNA14 overexpression group had a relatively slower growth rate and a better prognosis. Moreover, Notch1 was found to interact with GNA14 through IP/MS. Notch signals consist of receptors, ligands, translation regulators, and many other effectors 42, which have been reported to be involved in many tumors 43, 44. Accumulating evidence has shown that Notch1, a type of receptor, may inhibit the proliferation of HCC 45-47. NICD1 is the active form produced after Notch1 cleavage, which could inhibit inactivation of the RB pathway in HCC 28. Wang et al. reported that Notch1 signaling sensitized tumor apoptosis in HCC cells by inhibiting Akt/Hdm2-mediated p53 degradation and upregulating p53-dependent DR5 expression 48. Through immunofluorescence, we observed that GNA14 and Notch were co-localized in HCC cells. Co-immunoprecipitation and mass spectrometry detection further verified the relationship between GNA14 and Notch. Overexpression of GNA14 increased the expression of NCID1, RB, and cyclin p21. CCK8 and colony formation proved that Tangeretin (Notch1 inhibitor) can reverse the inhibitory effect of GNA14. Therefore, GNA14 could promote the cleavage of Notch1 and the production of NICD1, which contributed to the activation of the RB pathway, and ultimately inhibited HCC proliferation.

Tumor metastasis is another major feature of HCC. The invasion and migration ability of tumor cells overexpressing GNA14 were significantly inhibited in both* in vitro* and *in vivo* experiments. Through GCBI analysis, we identified JMJD6 as a potential interacting protein with GNA14. JMJD6, an Fe (II) - and 2-oxoglutarate (2OG) - dependent oxygenase, has been reported to be related to embryonic development, cell proliferation and migration, induction of self-tolerance in the thymus, and adipocyte differentiation 29, 49-51. The abnormal expression of JMJD6 is closely related to neuropathic pain, gestational diabetes mellitus, and various tumorigenesis 51. As an oncogene, JMJD6 was found to promote tumor cell invasion and migration, and accelerate tumor cell proliferation in breast cancer, melanoma, glioma, and HCC 29, 49, 52, 53 . In addition, it has been reported that GNA14 interacts with JMJD6 in HEK293 cells 54. When GNA14 was overexpressed in SK-Hep-1 cells, the expression of JMJD6 was downregulated. Co-IP assay and immunofluorescence further confirmed the interaction or correlation between GNA14 and JMJD6. When treated with CHX, the degradation rate of JMJD6 in the GNA14 overexpression group was faster, indicating that GNA14 may affect the JMJD6 protein level by regulating its stability. And when JMJD6 was overexpressed in SK-Hep-1 and Hep3B cells, it can reverse the effect of GNA14 on inhibiting HCC metastasis. Therefore, we speculated that GNA14 inhibited tumor invasion and migration by inhibiting JMJD6.

In summary, we screened the tumor suppressor GNA14 through combined database analysis and verified that GNA14 could inhibit HCC proliferation and metastasis both *in vitro* and* in vivo*. As a DNA methylation-related gene, GNA14 could be regulated by HBx by modulating the methylation status of its promoter. Therefore, GNA14 may be a potential biomarker and therapeutic target in HBV-related HCC.

## Supplementary Material

Supplementary figures and tables.

## Abbreviations

cccDNA: covalently closed circular DNA
CCK8: cell counting kit-8
DEGs: differentially expressed genes
ROC: receiver operating characteristic curve
DNMT: DNA methyltransferase
GNA: guanine nucleotide-binding protein subunit α
HBV: hepatitis B virus
HBx: HBV-encoded X protein
HCC: hepatocellular carcinoma
IHC: immunohistochemistry
IP: immunoprecipitation
JMJD6: Jumonji domain-containing 6
MS: mass spectrometry
NICD: Notch intracellular domain
SMART: shiny methylation analysis resource tool
siRNA: small interference RNAs
2-OG: 2-oxoglutarate
FC: fold change
FDR: false discovery rate
