# Supplementary material for: Hypermethylation of GNA14 and its tumor-suppressive role in hepatitis B virus-related hepatocellular carcinoma
Source: Theranostics. 2021 Jan 1;11(5):2318–33. doi: 10.7150/thno.48739 (PMC7797690; doi:10.7150/thno.48739)
Supplement: Supplementary file 1 — Supplementary figures and tables. [file thnov11p2318s1.pdf]

Supplementary Figure legend

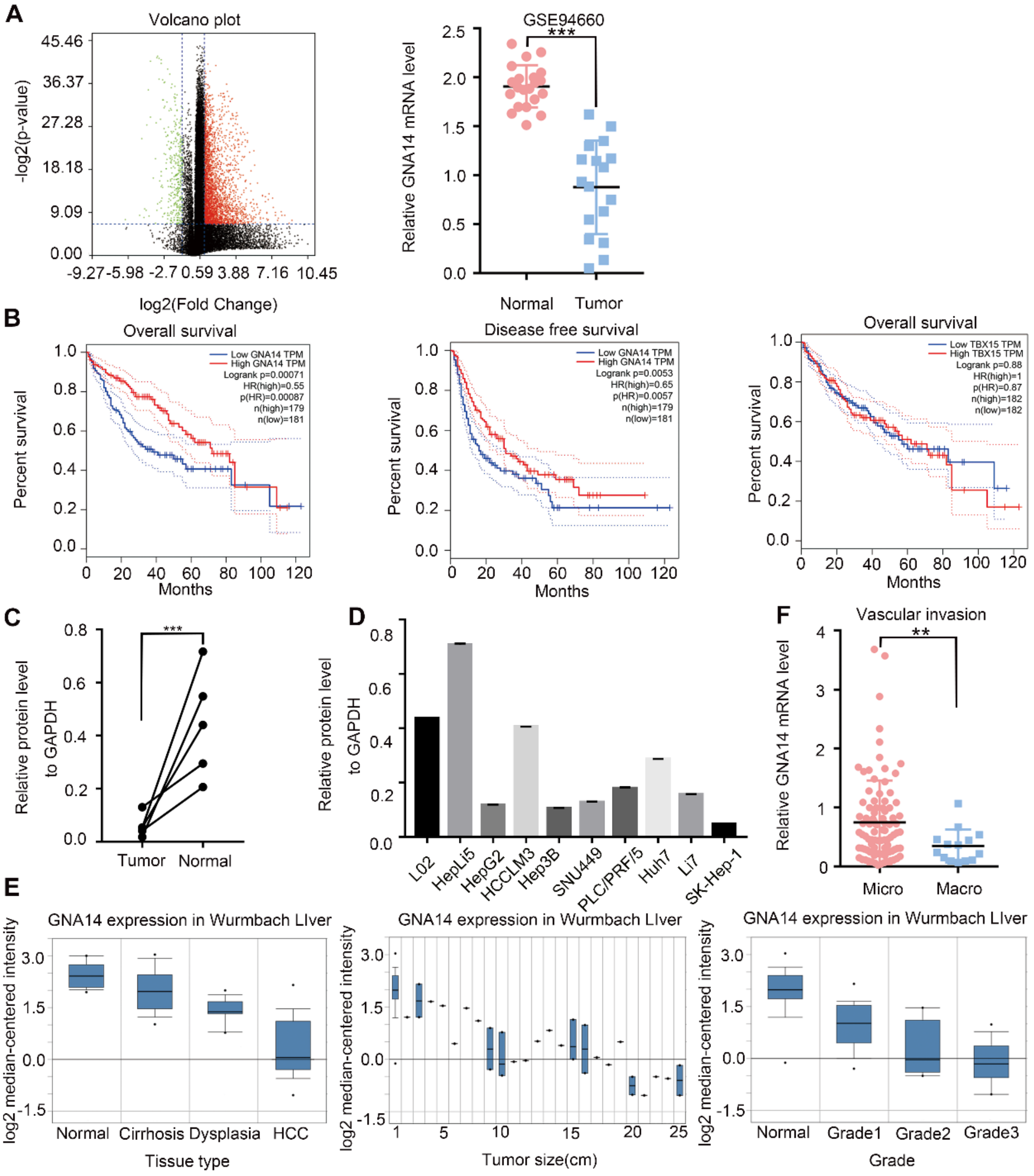

Supplement 1. GNA14 is downregulated in HCC. (A) Volcano plot of genome-wide gene expression profiles and GNA14 mRNA expression profiles in HCC and adjacent normal tissues from GSE94660. (B) Kaplan–Meier survival analysis of GNA14 and TBX15 in TCGA database. (C) Quantitative analysis of Western blot results of GNA14 expression in HCC tissues and matched normal tissues. (D) Quantitative analysis of Western blot results of GNA14 expression in HCC cell lines. (E) GNA14 mRNA levels in HCC patients with different tissue type, different tumor size and different tumor grade in Wurmbach Liver cohort

from omcomine database. (F) GNA14 mRNA levels in HCC patients with different vascular invasion in TCGA database. Statistical significance was determined by unpaired t test. Kaplan–Meier survival analysis was performed to analyze the survival percentage.

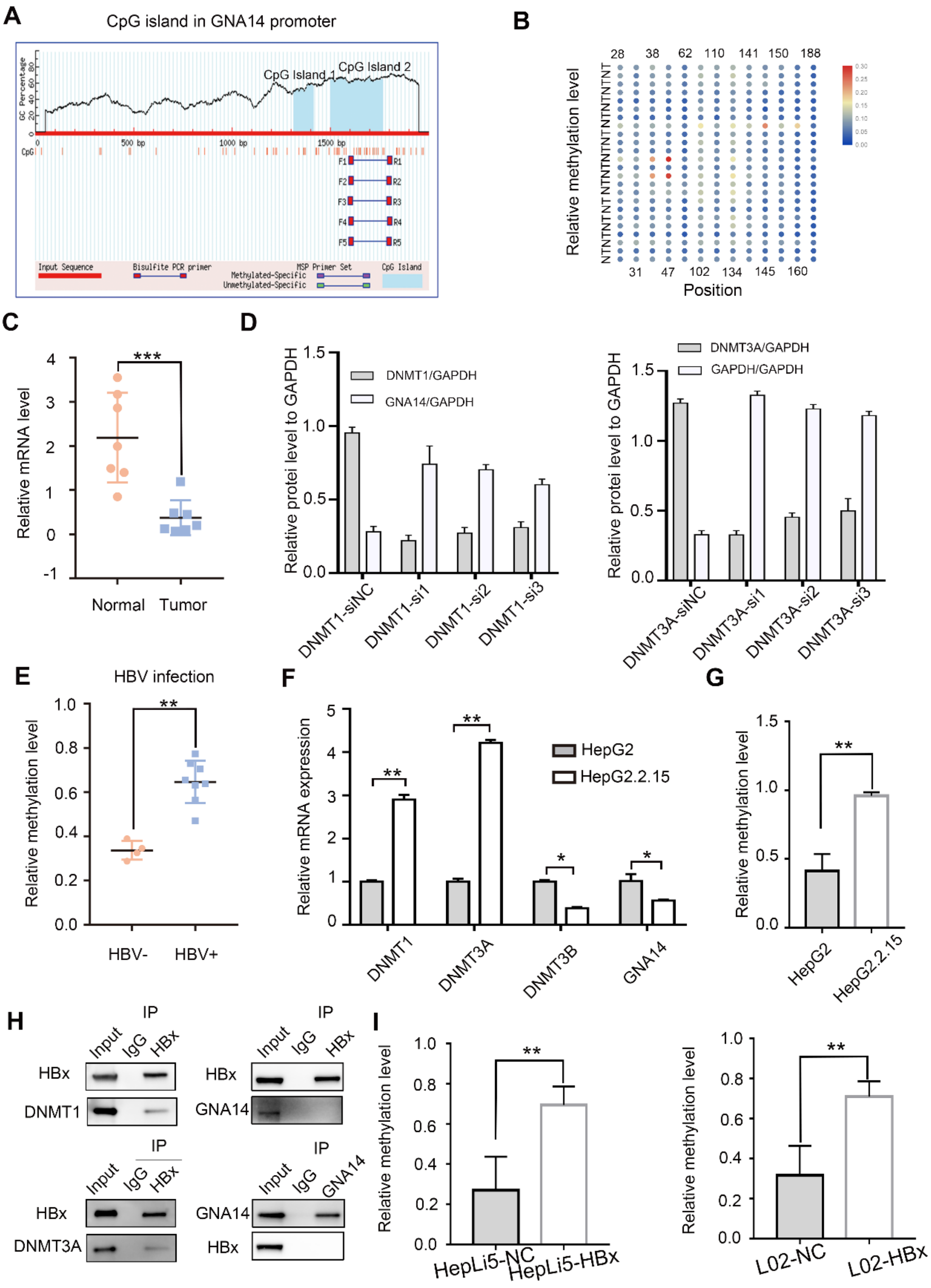

Supplement 2. GNA14 methylation is up-regulated in HCC and is regulated by HBx. (A) CpG islands in GNA14 promoter predicted by METHPRIMER. (B) The heatmap of the methyl target sequencing results indicated the methylation levels of 15 methylation sites in CpG island (Genomic position 2: 80263568-80263728) of the GNA14 promoter region in 12 pairs of matched tumor tissues and normal tissues in our hospital. “T” refers to tumor tissue and “N” refers to normal tissue. (C) Relative GNA14 mRNA level in 7 pairs of the tumor tissues and matched normal tissues used in Methyl-target DNA methylation sequencing analysis. (D) Quantitative analysis of Western blot results of SK-Hep-1 cells knocking down DNMT1 or DNMT3A. (E) Relative GNA14 methylation levels in HCC patients with or without HBV infection of Zheyi Hospital. Statistical significance was determined by unpaired t test. (F) The relative mRNA levels of DNMT1, DNMT3A and GNA14 in HepG2 and HepG2.2.15 cells. (G) DNA methylation sequencing of HepG2 and HepG2.2.15 cells in target CpG island. (H) The results of Co-IP were used to explore the combination of HBx and DNMT1 or DNMT3A or GNA14. (I) Results of DNA methylation sequencing after transfection of HBx overexpression plasmid in HepLi5 and L02 cells.

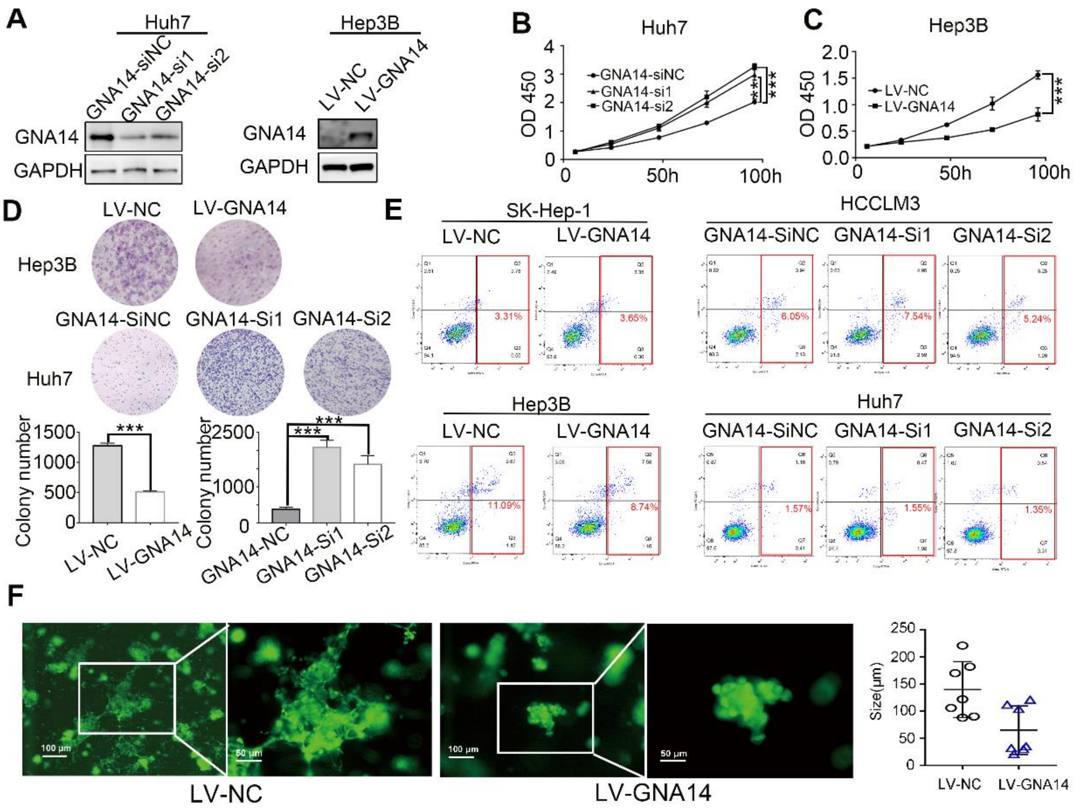

Supplement 3. GNA14 inhibits HCC proliferation. (A) Western blot was performed to verify the effect of GNA14 knockdown in Huh7 cells and overexpression in Hep3B cells. (B) CCK8 analysis was performed to determine the proliferation of Huh7 cells with GNA14 knockdown. (C) CCK8 analysis was performed to determine the proliferation of Hep3B cells infected with GNA14 lentivirus (LV-GNA14). (D) Colony assay of Hep3B cells infected with LV-GNA14 or LV-NC. And clone assay after knockdown of GNA14 in Huh7 cells. (E) Annexin V and propidium iodide staining experiments detected the apoptosis of HCC cells after overexpressing GNA14 (Hep3B and SK-Hep-1 cells) and knocking down GNA14 (HCCLM3 and Huh7 cells). (F) The effect of GNA14 overexpression on cell migration was examined by 3D cell culture.

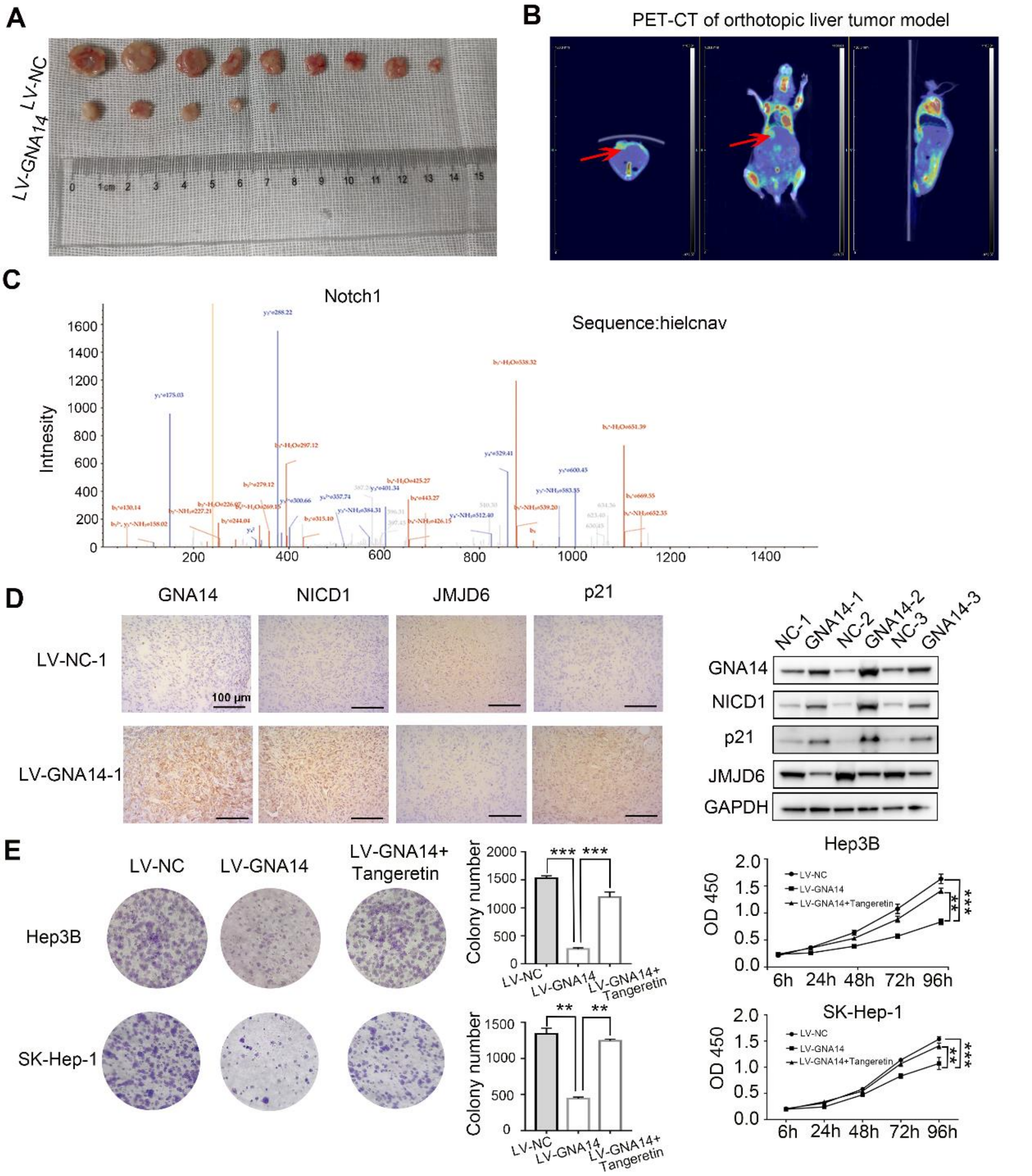

Supplement 4. GNA14 inhibits HCC proliferation through Notch1 pathway. (A) Xenograft tumors were generated by injecting SK-Hep-1 cells overexpressing GNA14 or carrying a control vector. (B) PET-CT images of nude mice orthotopic liver tumor model injected SK-Hep-1 cells overexpressing GNA14 or carrying a control vector. Red arrows indicated HCC. (C) Notch1 fragment detected by mass spectrometry is presented. (D) IHC staining and Western blot experiments of subcutaneous tumor were performed to study the expression of GNA14, NICD1, JMJD6 and P21. (E) Colony assay and CCK8 experiment were used to detect the proliferation of Hep3B and SK-hep-1 cells after

overexpression of GNA14 and treated with Tangeretin (Notch1 inhibitor).

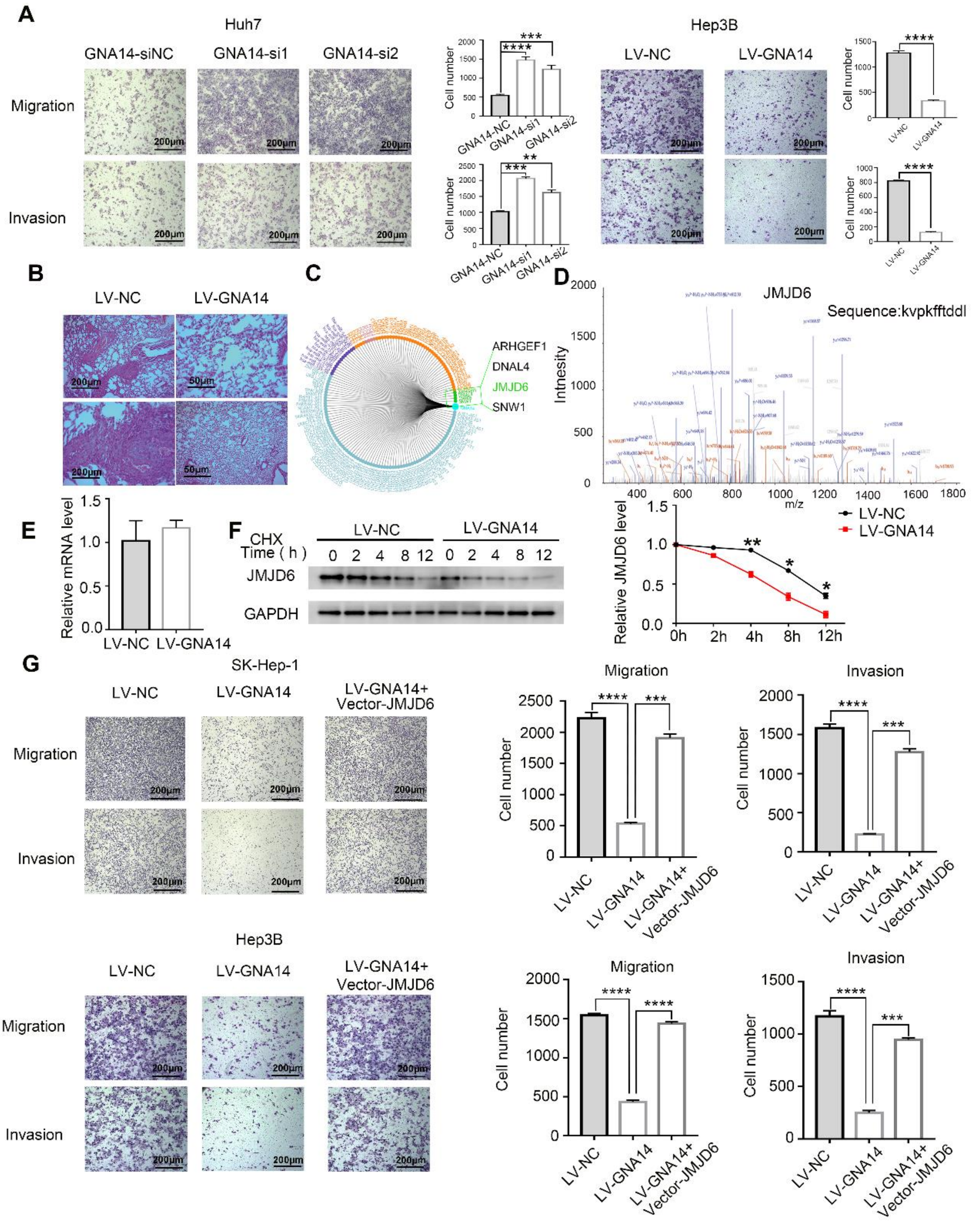

Supplement 5. GNA14 inhibits HCC invasion and migration through JMJD6. (A) The effects of knockdown of GNA14 in Huh7 and overexpression of GNA14 in Hep3B on cell migration and invasion were detected by transwell assay. (B) IHC image of lung in the lung colonization mouse model. (C) Potential interacting proteins with GNA14 analyzed by GCBI. (D) JMJD6 fragment detected by mass spectrometry is presented; b and y stand for N-terminal and

C-terminal collision-induced dissociation fragments. (E) The qPCR results of JMJD6 after overexpression of GNA14 in SK-Hep-1 cells. (F) The SK-Hep-1 cell line was treated with 10ug/ml CHX to detect the changes in JMJD6 protein expression at different treatment times. (G) The effect of JMJD6 overexpression on cell migration and invasion in SK-Hep-1 and Hep3B cells overexpressed GNA14.

Table S1

Sequences of siRNAs

Name	Sequence
HBx siRNA1	UCACCUCUGCACGUAGCAUTT;
HBx siRNA2	CCUUGAGGCAUACUUCAAATT;
HBx siRNA3	GAGGCUGUAGGCAUAAAUUTT;
HBx siRNA control	UUCUCCGAACGUGUCACGUTT.
GNA14-si1	TCACGAAGCTGGTTTACCA
GNA14-si2	CATGTACTCTCATCTAATT
DNMT1-si1	GAAGAGACGTAGAGTTACA
DNMT1-si2	GGAACTTTGTCTCCTTCAA
DNMT1-si3	CAATGAGACTGACATCAAA
DNMT3A-si1	CCACCAAAGCAGGCGATGA
DNMT3A-si2	CCACGACAGCGATGAGAGT
DNMT3A-si3	GCCTGGAGCCACCAGAAGA
siRNA control	TTCTCCGAACGTGTCACGT

Table S2

The primers for qRT-PCR

Name	Sequence
GNA14 forward	GCTGAGTGTGACAACGAGAAT

GNA14 reverse	TCCTGTTTCGGTCCTGTGTAT
GAPDH forward	GAGCCAAAAGGGTCATCATCT
GAPDH reverse	TTCCACGATACCAAAGTTGTCA
HBx forward	GCACTTCGCTTCACCTCT
HBx reverse	TATGCCTACAGCCTCCTA
DNMT1forward	GGAAGAAGACAAAGACCAGGAT
DNMT1 reverse	AGTTTCTGTTTGGGTGTTGGTT
DNMT3A forward	GAAAGGACGGAGAGGAGCA
DNMT3A reverse	ATGGATGGGGACTTGGAGAT

Table S3

Primary Antibodies

Name	Manufacturer	Catalog
GAPDH	CST	5174T
GNA14	Sigma	SAB1402481
HBx	abcam	AB157480
DNMT1	Santa Cruz	sc-271729
DNMT3A	Santa Cruz	sc-365769
Notch1	ThermoFisher	MA5-11961
NICD1	ThermoFisher	PA5-99448
P21	abcam	ab109520
RB	abcam	ab181616
JMJD6	Proteintech	16476-1-AP
